# Thermochemical Activity of Single- and Dual-Phase Oxide Compounds Based on Ceria, Ferrites, and Perovskites for Two-Step Synthetic Fuel Production

**DOI:** 10.3390/molecules28114327

**Published:** 2023-05-25

**Authors:** Alex Le Gal, Anne Julbe, Stéphane Abanades

**Affiliations:** 1 Processes, Materials and Solar Energy Laboratory (PROMES-CNRS), 7 Rue du Four Solaire, 66120 Odeillo Font-Romeu, France; 2 Institut Européen des Membranes (IEM), CNRS, ENSCM, University of Montpellier, Place Eugène Bataillon, 34095 Montpellier, France

**Keywords:** hydrogen, water-splitting, CO_2_ conversion, thermochemical cycles, concentrated solar energy, perovskite, oxygen-carrier redox materials, solar fuels, reticulated foams, composites

## Abstract

This study focuses on the generation of solar thermochemical fuel (hydrogen, syngas) from CO_2_ and H_2_O molecules via two-step thermochemical cycles involving intermediate oxygen-carrier redox materials. Different classes of redox-active compounds based on ferrite, fluorite, and perovskite oxide structures are investigated, including their synthesis and characterization associated with experimental performance assessment in two-step redox cycles. Their redox activity is investigated by focusing on their ability to perform the splitting of CO_2_ during thermochemical cycles while quantifying fuel yields, production rates, and performance stability. The shaping of materials as reticulated foam structures is then evaluated to highlight the effect of morphology on reactivity. A series of single-phase materials including spinel ferrite, fluorite, and perovskite formulations are first investigated and compared to state-of-the-art materials. NiFe_2_O_4_ foam exhibits a CO_2_-splitting activity similar to its powder analog after reduction at 1400 °C, surpassing the performance of ceria but with much slower oxidation kinetics. On the other hand, although identified as high-performing materials in other studies, Ce_0.9_Fe_0.1_O_2_, Ca_0.5_Ce_0.5_MnO_3_, Ce_0.2_Sr_1.8_MnO_4_, and Sm_0.6_Ca_0.4_Mn_0.8_Al_0.2_O_3_ are not found to be attractive candidates in this work (compared with La_0.5_Sr_0.5_Mn_0.9_Mg_0.1_O_3_). In the second part, characterizations and performance evaluation of dual-phase materials (ceria/ferrite and ceria/perovskite composites) are performed and compared to single-phase materials to assess a potential synergistic effect on fuel production. The ceria/ferrite composite does not provide any enhanced redox activity. In contrast, ceria/perovskite dual-phase compounds in the form of powders and foams are found to enhance the CO_2_-splitting performance compared to ceria.

## 1. Introduction

In the frame of the production of solar thermochemical fuels, two-step thermochemical cycles for splitting water or CO_2_ molecules offer several potential advantages. The first is the high theoretical energy conversion efficiency that can be achieved through the direct use of concentrated solar energy without the intermediate solar heat or photons conversion to electricity [1]. Solar thermochemical fuel production is indeed advantageous compared to electrochemical or photo(electro)chemical routes because it uses the entire solar spectrum for splitting H_2_O and CO_2_ molecules and, as such, provides a thermodynamically favorable path for efficient solar energy conversion and storage into fuels. Oxygen release and H_2_/CO production occur in different steps, avoiding their possible recombination and high-temperature gas separation. Moreover, the need for noble and costly metal catalysts is not required, and all the oxygen-carrier materials involved in the process are recycled. H_2_O and CO_2_ are the only inputs while concentrated solar energy is used as the high-temperature heat source for thermochemical reactions. Two-step thermochemical cycles involve a redox couple (M_x_O_y_/M_x_O_y−δ_), which allows the temperature to be reduced compared to the direct thermolysis of H_2_O or CO_2_ molecules. In the first step (Equation (1)), an oxide is thermally reduced under highly concentrated solar energy, thereby creating oxygen vacancies and releasing oxygen from the oxide lattice (at ~1400 °C). During the second step (Equation (2)), the activated oxide reacts with water vapor or CO_2_ to be re-oxidized and produce H_2_ or CO.

M_x_O_y_ → M_x_O_y−δ_ + δ/2 O_2_ (1)

M_x_O_y−δ_ + δH_2_O (or CO_2_) → M_x_O_y_ + δ H_2_ (or CO) (2)

Different types of relevant metal oxide redox cycles have been considered. They chiefly include volatile (such as ZnO/Zn, SnO_2_/SnO, MgO/Mg) or non-volatile oxides (such as Fe_3_O_4_/FeO and ferrites, CeO_2_/CeO_2−δ_ and substituted ceria, and ABO_3_/ABO_3−δ_ perovskites systems). The first category involves simple oxides with discrete transitions in the metal oxidation state (leading to high oxygen amounts being released and recovered during each cycle) and thus produces high fuel amounts per mass of active material at the expense of recombination issues with O_2_ during the reduction step. The second category (that chiefly involves non-stoichiometric oxides) does not exhibit any phase change while O_2_ is released from the solid, thus limiting the recombination and avoiding the need for the oxide transport between steps. However, they feature a lower mass-specific fuel productivity than the stoichiometric oxides due to a lower amount of oxygen being exchanged during solid-state reactions (quantified by the magnitude of oxygen non-stoichiometry δ). Currently, three main classes of redox-active non-stoichiometric compounds are considered to act as intermediate redox materials. Ferrites, ceria, and perovskite-based oxides have the ability to be partially reduced and oxidized reversibly without any phase changes (solid state) during redox reactions, maintaining their stable crystallographic structure. They feature a continuous redox activity over changes in temperature and oxygen partial pressure, thanks to the creation of oxygen vacancies that facilitate ionic diffusion. Nickel ferrites (NiFe_2_O_4_) were first identified as the most promising iron-based oxides for thermochemical cycles [2,3]. A large reduction yield was achieved at 1400 °C, and a good water-splitting capacity of the reduced species was demonstrated. The main drawback of this oxide is the low thermal stability after several cycles. The material tends to sinter and in turn, the reactivity decreases over cycles. Several solutions have been considered to improve thermal stability, such as supporting Ni-ferrites on ZrO_2_ support [4,5] or using ferrite powder in a fluidized bed reactor to limit particle growth and promote heat and mass transfer [6]. Ceria and doped ceria have also been widely investigated for use in thermochemical redox cycles based on an initial study in 2006 [7,8,9,10]. Pure ceria demonstrated fast redox kinetics and good thermal stability during cycling, but the process is limited by a relatively low fuel productivity per gram of material due to the low reduction extent δ. High temperatures and/or low partial pressures of O_2_ during the reduction step are additionally required to enhance δ. Alternatively, ceria doping with cations (such as Zr^4+^) can increase the reduction efficiency and fuel productivity at the expense of a decrease in oxidation kinetics [11,12]. Another way to improve material reactivity and kinetics is to use materials with designed morphologies and microstructures [13], such as reticulated porous structures [14,15,16], 3D-ordered macroporous structures [17,18], fibers [19,20], and porous microspheres [21]. A third family of oxides actively studied consists of the perovskite oxides for their capacity to be thermally reduced while maintaining their crystalline structure stable and to adapt to a large space of compositions. This great versatility permits to design and optimize materials with improved thermochemical performance. Manganite perovskites such as La_0.5_Sr_0.5_MnO_3_ [22,23], BaCe_0.25_Mn_0.75_O_3_ [24], or La_0.8_Sr_0.2_(Mn_0.2_Fe_0.2_Co_0.4_Al_0.2_)O_3_ [25] have shown attractive fuel production yields per cycle although they are still limited by kinetics.

Although these materials have shown their ability to be used in two-step solar thermochemical cycles, an improvement in the overall solar-to-fuel efficiency is still expected. Improving the performance of materials is the main lever for success among the different possibilities such as the tuning of process conditions or the optimization of reactor design [26,27]. Indeed, even a small increase in material productivity will have a strong impact over thousands of cycles day after day.

Most recent studies have focused on materials with spinel, perovskite, and fluorite structures as redox catalysts (oxygen carriers) in this process [28]. To date, very little research has considered a comparison of the thermochemical performance of redox materials based on such structures. In addition, the activity of dual-phase compounds has been scarcely studied so far. For this purpose, experimental studies are required to evaluate the reactivity of various compounds applied to thermochemical cycles and to identify the most suitable compositions. In this work, different formulations of redox-active materials were synthesized, characterized, and tested during thermochemical cycles based on various structural configurations and cationic substitutions in the crystal lattice of the materials, with the aim of comparing their performance with state-of-the-art materials. Additionally, their redox activity has been studied by focusing on their ability to perform the splitting of CO_2_ or H_2_O molecules during thermochemical cycles while quantifying the fuel production yields, rates, and performance stability.

The thermochemical activity and stability of materials with different structures and formulations were compared under the same cycling conditions. The evolution of the reactivity from one cycle to another was discussed. Different materials’ morphologies were also considered to show possible effects on fuel production yields.

First, the performance of nickel ferrite and ceria shaped as reticulated porous structures (open-cell foams) was compared, as they represent the state-of-the-art materials for this application. Then, other formulations were considered based on recent studies reported in the literature. Previous works indeed identified different new formulations such as Ce_0.9_Fe_0.1_O_2_, Ca_0.5_Ce_0.5_MnO_3_, Ce_0.2_Sr_1.8_MnO_4_, and Sm_0.6_Ca_0.4_Mn_0.8_Al_0.2_O_3_ as attractive candidates for two-step redox cycles, but some of them reported unusual performance in comparison to benchmark materials, such as ceria, ferrites, or lanthanum manganite perovskites. It is therefore necessary to carry out additional experimental tests to check whether such materials are really promising. These materials were thus selected in this work to assess and compare their redox activity under given operating conditions commonly used in thermochemical cycles and which are typically required for the splitting of the CO_2_ molecule. Finally, dual-phase materials consisting of ceria/ferrite and ceria/perovskite composites were considered to determine whether adding a phase more reducible than ceria can improve overall fuel production.

## 2. Results and Discussion

### 2.1. Ni-Ferrite (NiFe_2_O_4_) Foam

**Figure 1:**
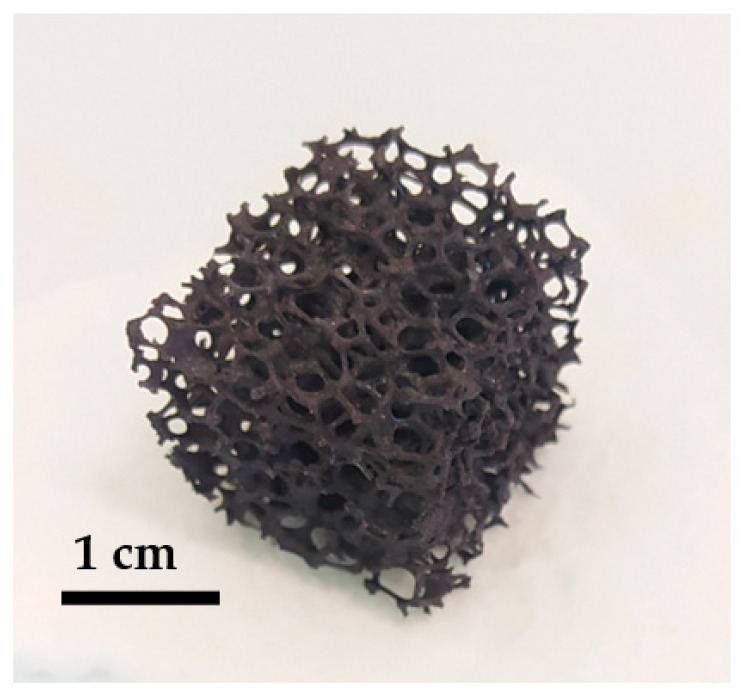
Picture of a pristine NiFe_2_O_4_ foam calcined at 1400 °C.

NiFe_2_O_4_ has been widely studied for application in two-step solar thermochemical cycles [29,30,31], and this work investigates the reactivity of nickel ferrite foam in order to compare it to ferrite powder and ceria foam. The nickel ferrite foam was obtained by the replication method detailed in the Materials and Methods section. Figure 1 shows a picture of the foam after calcination at 1400 °C (before redox cycles).

**Figure 2:**
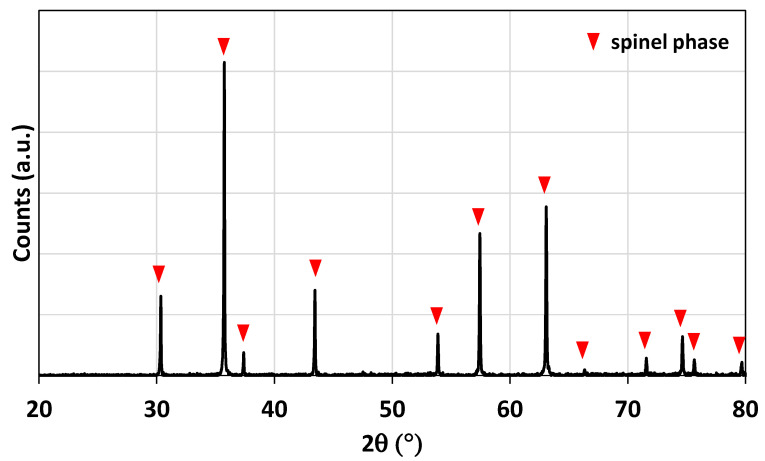
X-ray diffraction pattern of the NiFe_2_O_4_ foam after calcination at 1400 °C.

The ferrite foam was characterized by X-ray diffraction. The diffraction pattern of the crushed foam (before redox cycles) is shown in Figure 2. The spinel structure associated with the doped ferrite is observed.

**Figure 3:**
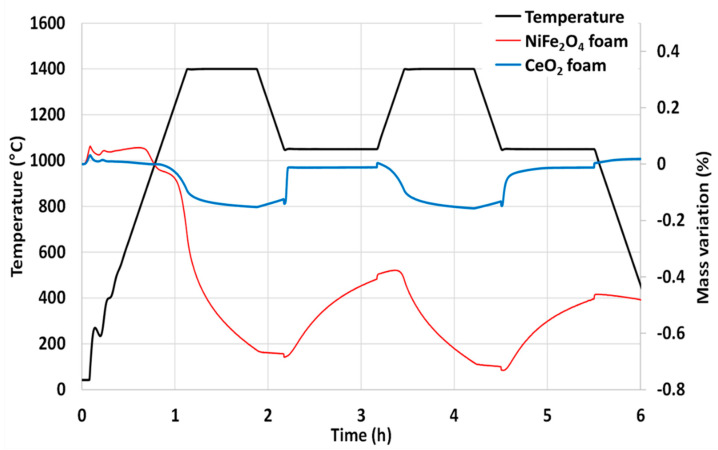
Thermogravimetric analysis of two consecutive CO_2_-splitting cycles with nickel ferrite and ceria foams.

The nickel ferrite foam was also characterized by thermogravimetric analysis (TGA) during two consecutive CO_2_-splitting cycles and further compared with ceria foam (Figure 3). The mass loss of the sample is due to the oxygen released from the material during the thermal reduction. The increase in mass is due to the oxygen uptake by the material during the supply of CO_2_ (oxidant). The negative value of mass loss means that the mass of the sample is decreased from its initial mass due to the release of oxygen. It was found that 201 µmol of O_2_ per gram of NiFe_2_O_4_ was released during the first reduction step at 1400 °C and 172 µmol of CO per gram was produced during the first oxidation step with a peak production rate of 6.2 µmol_CO_/min.g (measured a few seconds after CO_2_ injection). The second cycle produced 106 µmol of O_2_ per gram during the reduction step and 158 µmol of CO per gram during the oxidation step with a peak production rate of 6.2 µmol_CO_/min.g. These values are consistent with previously reported data [32]. Compared to the CeO_2_ foam yielding about 100 µmol_CO_/g under the same conditions, the nickel ferrite foam produces much more CO but a slight decrease in reactivity is observed during cycling, contrary to ceria. This well-known phenomenon is explained by the limitations due to sintering and kinetic effects for ferrites, while their shaping as a porous foam does not alleviate this phenomenon.

### 2.2. Iron-Doped Ceria (Ce_0.9_Fe_0.1_O_2_)

**Figure 4:**
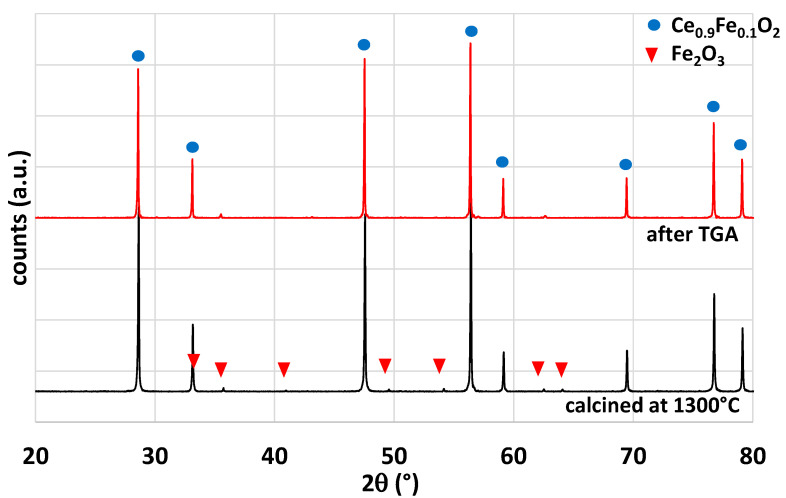
Comparison of the X-ray diffraction patterns for Ce_0.9_Fe_0.1_O_2_ after calcination at 1300 °C and after TGA analysis.

Iron-doped ceria powder has been investigated during CO_2_-splitting thermochemical cycles. This material was selected in this study because it was reported in the literature as a promising material for solar thermochemical fuel production [33,34]. Ce_0.9_Fe_0.1_O_2_ was synthesized by the coprecipitation method detailed in the Materials and Methods section. The powder was calcined at 1300 °C for 1 h (this temperature was selected to alleviate the effect of sintering). On leaving the oven, the powder was partially sintered and thus ground in a mortar. The powder was characterized by XRD (Figure 4). Two phases were observed after calcination at 1300 °C: a major phase related to the crystallographic structure of ceria (fluorite) and a minor phase of Fe_2_O_3_. The cubic lattice parameter of the fluorite phase was calculated from the diffraction peaks and a value of a = 5.400 (9) Å was found. Compared to pure ceria (a = 5.4112 Å [35]), a lower value was obtained for parameter a, which confirms the insertion of the iron atoms in the structure due to a smaller ionic radius. After cycles in TGA, the structure remained unchanged.

**Figure 5:**
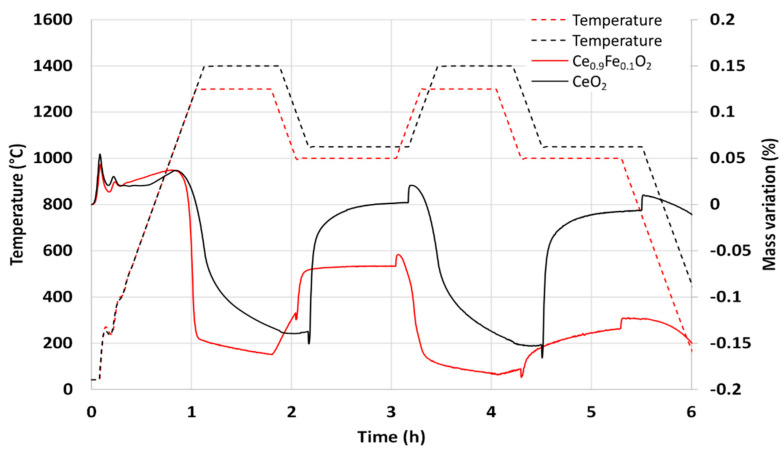
Thermogravimetric analysis of Ce_0.9_Fe_0.1_O_2_ during two consecutive CO_2_-splitting thermochemical cycles. The TGA curve obtained for ceria is reported for comparison.

Two consecutive thermochemical cycles were performed in TGA. Figure 5 shows the mass variation during the CO_2_-splitting cycles compared to pure ceria powder as a reference. The reduction temperature was 1300 °C (the same as the calcination temperature), and the oxidation step was carried out at 1000 °C. A steep mass loss was observed during the first reduction step associated with the reduction of Ce_0.9_Fe_0.1_O_2_, corresponding to an O_2_ release of 62 µmol/g. The re-oxidation was not complete during the first cycle with a CO production of only 36 µmol/g with a peak production rate of 15.1 µmol_CO_/min.g. During the second cycle, an O_2_ release of 40 µmol/g and a CO production of 33 µmol/g were measured with a peak production rate of 6.3 µmol_CO_/min.g. Compared to pure ceria, the reduction yield at 1300 °C was higher with faster kinetic rate, but the material undergoes strong sintering during thermal cycling, which leads to poor cyclability with a decrease in the reactivity. These results show different trends from those previously reported. Al-Taweel et al. [34] reported an H_2_ production 1.8 times greater than ceria after a reduction step at 1550 °C, while Orfila et al. [33] mentioned an H_2_ production of 8.5 STPcm^3^/g (380 µmol/g) with Ce_0.9_Fe_0.1_O_2_ powder after a reduction step at 1300 °C. The sintering issue was not reported as limiting the overall production performance of the cycle, and such high production yields are unexpected given the low thermal stability induced by iron doping. These results are therefore very different from each other and very far from the values found in this study.

### 2.3. Perovskites

**Figure 6:**
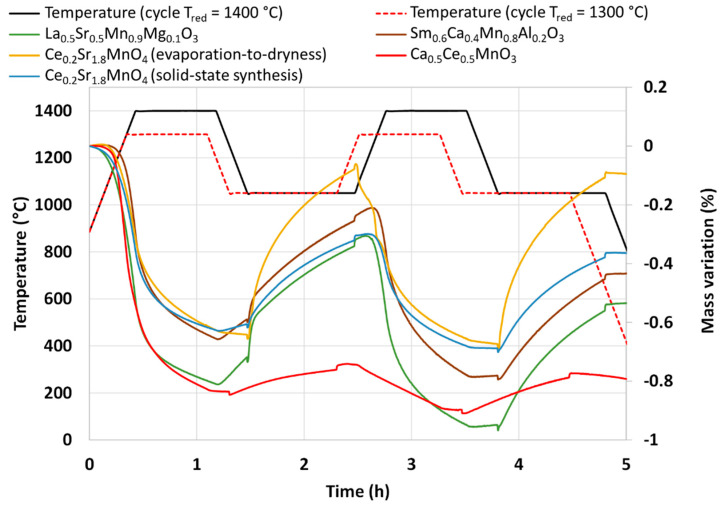
Thermogravimetric analyses of different perovskites during two consecutive CO_2_-splitting thermochemical cycles (note that the reduction step for Ca_0.5_Ce_0.5_MnO_3_ was carried out at 1300 °C).

Several perovskite compositions were also synthesized and characterized during thermochemical cycles. Table 1 presents the synthesis method, the powder formulations, and their reactivity during CO_2_-splitting cycles. La_0.5_Sr_0.5_Mn_0.9_Mg_0.1_O_3_ (LSMMg) is used as a reference material for the performance comparison as it was previously identified as a promising candidate material for such an application [36]. The thermogravimetric analyses are presented in Figure 6. Two consecutive CO_2_-splitting cycles were carried out with a reduction temperature of 1400 °C (except for Ca_0.5_Ce_0.5_MnO_3_ which was reduced at 1300 °C due to a melting issue above this temperature) and a re-oxidation temperature of 1050 °C.

LSMMg released 254 and 203 µmol/g of O_2_ during the reduction steps and produced 246 and 249 µmol/g of CO during the re-oxidation steps.

**Figure 7:**
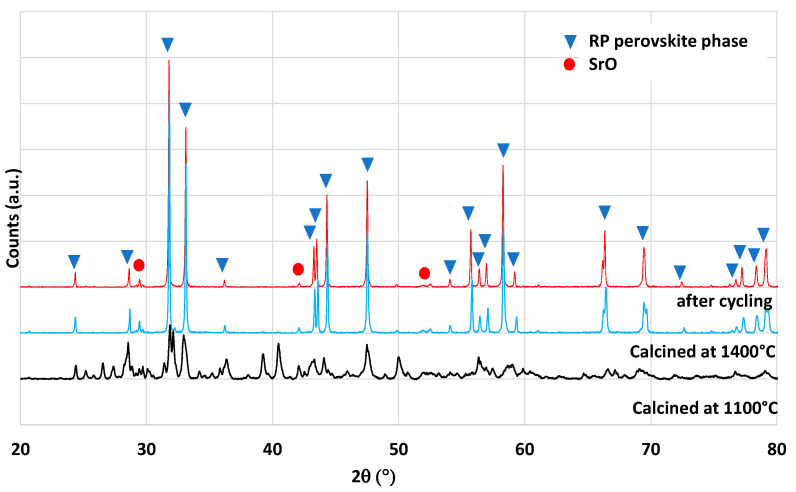
XRD pattern of Ce_0.2_Sr_1.8_MnO_4_ obtained from the evaporation-to-dryness method.

**Figure 8:**
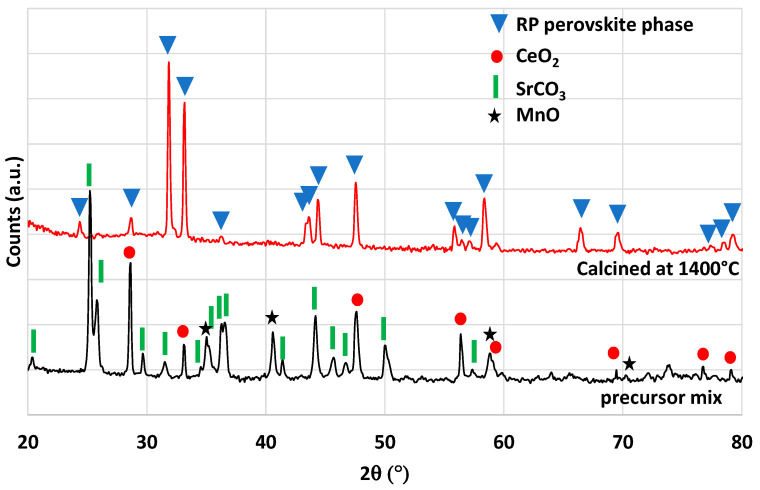
XRD patterns of Ce_0.2_Sr_1.8_MnO_4_ obtained at 1400 °C by solid-state synthesis. The starting mixture of precursors is reported for comparison.

The Ruddlesden–Popper (RP) perovskite Ce_0.2_Sr_1.8_MnO_4_ was identified by Barcellos et al. from thermodynamic calculations as a good candidate for the production of synthetic fuel via thermochemical cycles [37]. Bergeson-Keller et al. experimentally tested this material during water-splitting cycles [38]. In this work, Ce_0.2_Sr_1.8_MnO_4_ was synthesized by a soft synthesis method (evaporation-to-dryness method) and a solid-state mechanical method both described in the Materials and Methods section. The material synthesized by the Pechini method produced 201 and 114 µmol/g of O_2_ and 219 and 224 µmol/g of CO from mass losses measured by TGA. XRD analysis (Figure 7) reveals the presence of a SrO phase that could react with CO_2_ to produce SrCO_3_ [39]. This side reaction participates in the mass increase during the re-oxidation step, and this is confirmed during the second reduction step by a first mass decrease just after the dwell at 1050 °C when the CO_2_ injection was stopped. This is attributed to the strontium carbonate dissociation releasing CO_2_. A second synthesis method was used to obtain the pure RP perovskite phase without a SrO secondary phase (solid-state synthesis), and the material was tested in the same way with the thermobalance. The XRD analysis confirms the absence of a SrO phase (Figure 8). Accordingly, the mass variations were lower during TGA. The O_2_ release was 197 and 121 µmol/g and the CO production was 184 and 199 µmol/g. The previous study found in the literature [38] reported an O_2_ release at 1400 °C of 168 and 142 µmol/g and a hydrogen production of 247 µmol/g at 1000 °C with a 40%_vol_ steam concentration. The material obtained in this study thus released more O_2_ during the first reduction, but the CO yield was hindered by kinetic limitations. Note that a longer duration for the re-oxidation step would result in higher fuel production, since the oxidation step with CO_2_ did not reach completion after 45 min at 1050 °C. This investigation also reveals the importance of the synthesis method on materials’ performance.

**Figure 9:**
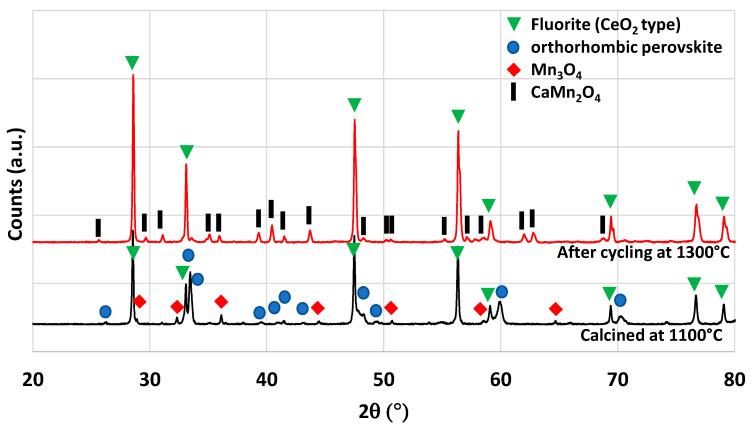
XRD patterns of Ca_0.5_Ce_0.5_MnO_3_ calcined at 1100 °C and after thermochemical cycling at 1300 °C.

Another material formulation that was synthesized is Ca_0.5_Ce_0.5_MnO_3_. This perovskite composition was previously identified by theoretical calculations as a potential candidate with improved reactivity for thermochemical cycles [40]. The authors of this work mentioned the ability of simultaneous reduction of Ce^4+^ and Mn^3+^. However, it was never tested experimentally to confirm the theoretical predictions. The material was thus synthesized by the modified Pechini method (see the Materials and Methods section). The XRD pattern of the synthesized powder is shown in Figure 9. After calcination at 1100 °C, two main phases are observed, the fluorite structure associated with CeO_2_ and the perovskite phase associated with (Ca,Ce)MnO_3_. A minor phase of Mn_3_O_4_ is also observed on the XRD pattern. This observation is consistent with a previous study on Ca_0.5_Ce_0.5_MnO_3_ synthesis, where CeO_2_ was found to be a major phase obtained [41]. Hence, this material is a multi-phase material and the pure Ca_0.5_Ce_0.5_MnO_3_ phase was not obtained. The variation of mass during redox cycles from TG analysis is presented in Figure 6. Due to melting phenomena occurring at 1400 °C, the two consecutive cycles were performed with a reduction temperature of 1300 °C. During redox cycling, 260 µmol/g and 48 µmol/g of O_2_ were released, while 53 µmol/g and 76 µmol/g of CO were produced, respectively. At the end of the thermochemical cycles, the recovered powder was strongly sintered. Such performance does not overpass the gas production yields of ceria during CO_2_-splitting cycles after a reduction step at 1300 °C. XRD analysis after thermochemical cycling is shown in Figure 9, which reveals the formation of a CaMn_2_O_4_ phase instead of the perovskite phase that has totally disappeared after thermochemical cycles.

**Figure 10:**
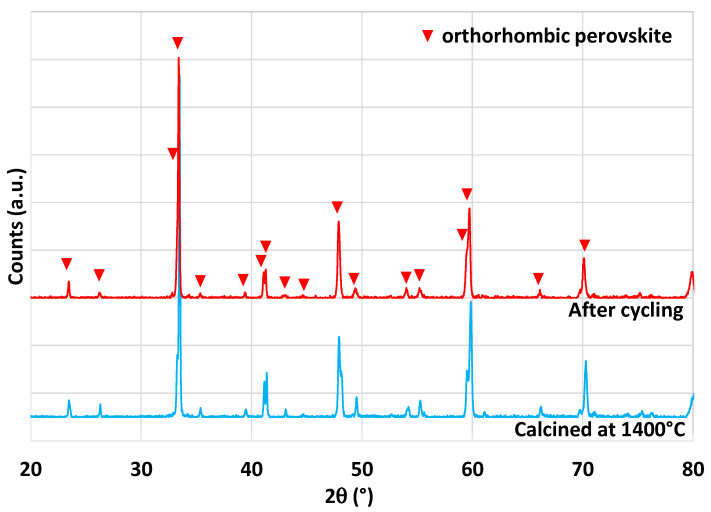
XRD patterns of Sm_0.6_Ca_0.4_Mn_0.8_Al_0.2_O_3_ prepared at 1400 °C and after thermochemical cycling at 1400 °C.

Another perovskite formulation considered in this study is Sm_0.6_Ca_0.4_Mn_0.8_Al_0.2_O_3_. This material was identified as highly promising during an experimental study [42], in which authors reported a very high and unusual CO production yield of 580 µmol/g over 14 consecutive cycles with a reduction step at 1350 °C. Thus, a complementary analysis of this material was performed in this work to compare results and discuss the performance. The material was synthesized by the modified Pechini method (see the Section 3). XRD analysis is shown in Figure 10. The orthorhombic perovskite phase associated with the Sm_0.6_Ca_0.4_Mn_0.8_Al_0.2_O_3_ phase was identified after calcination at 1400 °C. After cycling, the perovskite phase remained stable. During the two consecutive CO_2_-splitting cycles realized in the thermobalance, the O_2_ release was 206 and 180 µmol/g and the CO production yield reached 213 and 212 µmol/g. These values are thus far from those reported previously [42] and even lower than those of LSMMg used as reference formulation (Table 1). However, due to the slow kinetic rate, the oxidation step with CO_2_ did not reach completion after 45 min at 1050 °C, regardless of the considered perovskite material. Hence, a longer duration for the re-oxidation step would result in higher fuel production (the maximum CO production yield would however not exceed twice the O_2_ yield). Finally, this study confirms that the previously published unusual or unexpected data (e.g., related to Ce_0.9_Fe_0.1_O_2_ [33,34], Ca_0.5_Ce_0.5_MnO_3_ [40], or Sm_0.6_Ca_0.4_Mn_0.8_Al_0.2_O_3_ [42]) are difficult to reproduce experimentally and therefore strongly require experimental validation and confirmation.

### 2.4. Dual-Phase Composite Materials

Dual-phase materials were investigated to provide insights into the potential synergistic effect that may arise by mixing two redox-active materials with noticeable performance in thermochemical CO_2_-splitting cycles. The activity of dual-phase compounds has been scarcely studied so far. Only a few articles reported the benefits of dual-phase materials containing ceria on the oxygen storage/exchange capacity [43,44,45,46]. In this study, the mixture of ceria with nickel ferrite was studied, as well as the mixture of ceria with a perovskite (LSMMg). These composite materials are expected to offer enhanced reactivity in two-step thermochemical cycles, compared to individual compounds.

#### 2.4.1. Dual-Phase CeO_2_/NiFe_2_O_4_

**Figure 11:**
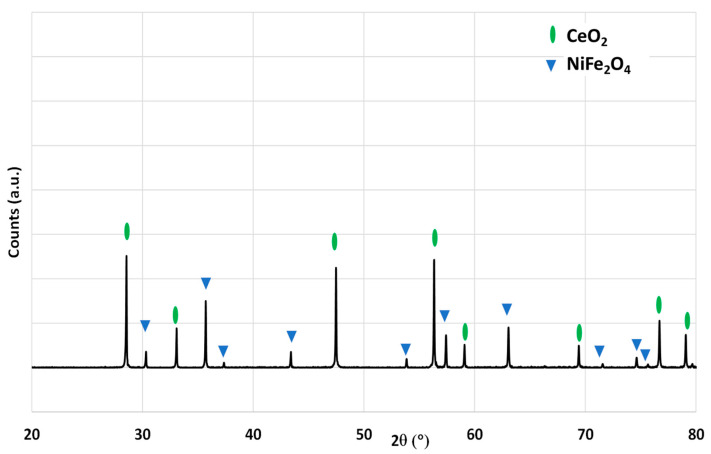
XRD pattern of a mixture of CeO_2_ and NiFe_2_O_4_ (50/50 wt%) after thermal treatment in air at 1400 °C.

A mixture of ceria and nickel ferrite (50/50 wt%) was obtained by grinding a commercial micrometric powder of cerium oxide (Sigma-Aldrich, St. Louis, MO, USA, 99.9%, <5 µm powder) with a previously synthesized nickel ferrite powder (obtained by the coprecipitation of hydroxides, see the Materials and Methods section) followed by calcination of the blend at 1400 °C under static air for 1 h. This thermal treatment permits to characterize the stability of dual-phase materials and to observe whether they react together at the temperature of the reduction step. The XRD pattern of the mixture after calcination is shown in Figure 11. The two distinct phases of ceria (fluorite) and nickel ferrite (spinel) are observed and no by-product has thus been formed, which confirms the thermal stability in air at 1400 °C.

**Figure 12:**
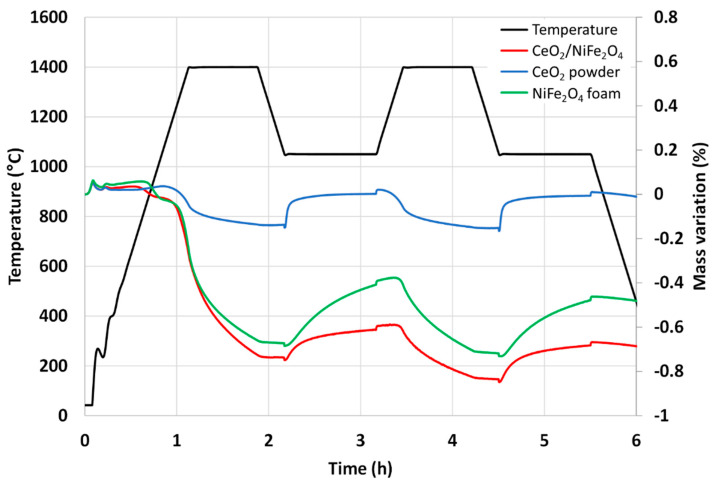
Thermogravimetric analysis of CeO_2_/NiFe_2_O_4_ mix (50/50 wt%) during two-consecutive CO_2_-splitting thermochemical cycles, in comparison with single phases of NiFe_2_O_4_ and CeO_2_.

The composite material was analyzed by TGA (Figure 12). During the two consecutive cycles, it released 226 µmol/g and 76 µmol/g of O_2_ during the reduction steps and produced 85 µmol/g and 104 µmol/g of CO during the oxidation steps (Table 2). Compared to the pure components, the dual-phase material did not show improved performance. The release of O_2_ was better than for pure ceria but lower than for pure Ni-ferrite, and the CO production yield was similar to that of ceria but lower than that of Ni-ferrite.

**Figure 13:**
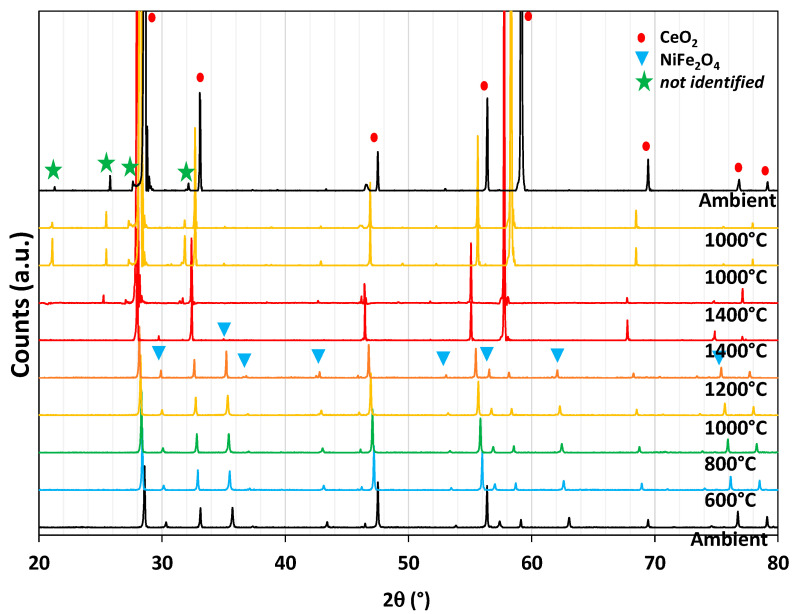
In situ XRD analysis of CeO_2_/NiFe_2_O_4_ (50/50 wt%) during a thermochemical cycle under a controlled atmosphere (N_2_ from ambient to 1400 °C and CO_2_ at 1000 °C).

XRD analysis of the mixture at different temperatures under a controlled atmosphere is shown in Figure 13. At the bottom, the pattern was measured at room temperature. Going up from bottom to top, the powder was heated under a flow of nitrogen up to 1400 °C, with patterns acquisition at 600 °C, 800 °C, 1000 °C, 1200 °C, and 1400 °C. A plateau at 1400 °C for 1.5 h preceded a drop in temperature to 1000 °C at which pure CO_2_ was swept inside the analysis chamber to react with the material. As a result, both ceria and Ni-ferrite phases are observed from room temperature to 1200 °C. The spinel phase corresponding to NiFe_2_O_4_ disappears at 1400 °C, and a new phase not identified appears at the end of the reduction step. This means the ferrite phase mixed with ceria is not stable under inert gas at 1400 °C, whereas the same mixture was stable during thermal treatment in air (Figure 11). During cooling as well as during the re-oxidation step, the spinel phase does not reappear. The two materials composing the mixture thus react together during heating at 1400 °C under an inert atmosphere, and the reactivity is not improved. Therefore, the investigations on this composite material were stopped and another composite material was studied.

#### 2.4.2. Dual-Phase CeO_2_/Perovskite

**Figure 14:**
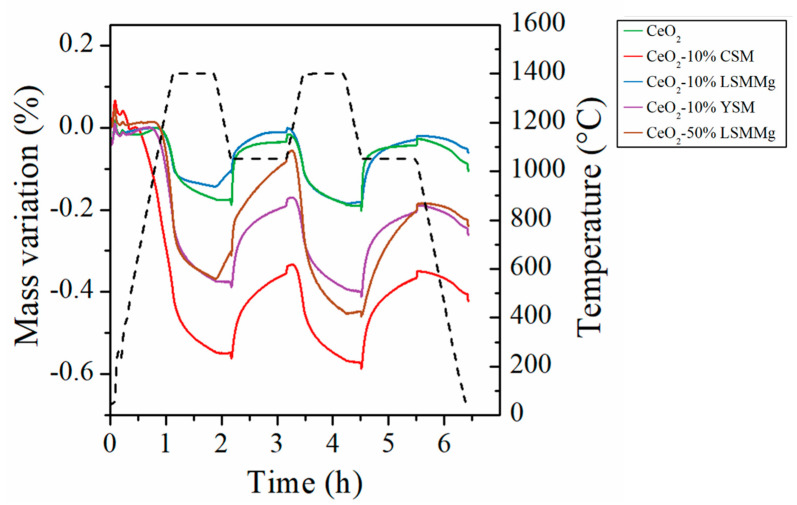
Thermogravimetric profiles (solid lines) along with the temperature profile (black dotted line) during thermochemical cycling of powder mixtures: CeO_2_-10% CSM, CeO_2_-10% LSMMg, CeO_2_-10% YSM, and CeO_2_-50% LSMMg, in comparison with single CeO_2_.

Some studies have already mentioned the use of dual-phase ceria/perovskite materials in thermochemical cycles with expected benefits on fuel production performance [43,47]. The objective of this study on dual-phase materials is to observe whether a synergistic phenomenon can improve performance compared to single compounds, in particular by maintaining a good reduction yield (property of perovskites) combined with increased re-oxidation kinetics (property of ceria). In order to study the redox behavior of ceria/perovskite dual-phase compositions, TGA was performed with ceria–perovskite powder mixtures (Figure 14). Five mixture compositions were studied: ceria powder mixed with 10 wt% of La_0.5_Sr_0.5_Mn_0.9_Mg_0.1_O_3_ (LSMMg), 50 wt% of LSMMg, 10 wt% of Ca_0.5_Sr_0.5_MnO_3_ (CSM), 50 wt% of CSM, and 10 wt% of Y_0.5_Sr_0.5_MnO_3_ (YSM as an alternative material to CSM), abbreviated as CeO_2_-10% LSMMg, CeO_2_-50% LSMMg, CeO_2_-10% CSM, CeO_2_-50% CSM, and CeO_2_-10% YSM, respectively. The O_2_ production yield was increased for CeO_2_-10% YSM, CeO_2_-50% LSMMg, CeO_2_-10% CSM, and CeO_2_-50% CSM in comparison with pristine ceria (Table 3). Accordingly, CO production yield was also increased, highlighting the beneficial impact on the redox activity of dual-phase compositions. Note that the CeO_2_-50%CSM material releases a large amount of O_2_ (652 µmol/g in the first cycle) due to the large amount of CSM phase which is easily reduced (it is thus not represented in Figure 14). However, such a high reduction yield does not improve the CO production capacity of the material to a great extent because the re-oxidation step is not complete (151 and 168 µmol/g in the first and second cycles, respectively). The same behavior is observed for the CeO_2_-10% CSM material.

**Figure 15:**
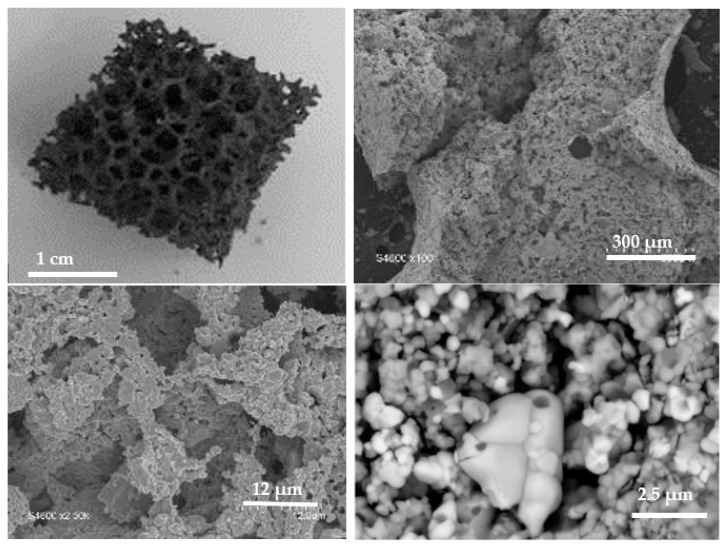
Macroscopic and microscopic images of the CeO_2_/LSMMg (50/50 wt%) dual-phase foam after calcination at 1400 °C.

In order to optimize the reactivity of the most interesting dual-phase material, a reticulated foam of CeO_2_/LSMMg (50/50 wt%) was prepared by the impregnation method after calcination of the powder mixture at 1400 °C in air (see the Materials and Methods section). Figure 15 presents the macroscopic and microscopic images of the foam. The foam exhibits a dark gray color due to LSMMg. The strands are highly porous and have a thickness of about 1 mm. Pores around grains are observed and the two materials are well mixed with a micrometric grain size. The foam thus exhibits a dual-scale porosity (millimetric cells and submicrometric pores within struts).

Actually, the specific surface area of the synthesized materials is always low after their thermal treatment at 1400 °C (<1 m^2^/g), which means that this property is not the main one impacting the CO yield. The porous structure of the foam can be advantageous because the most important is to keep good access of the reactive gas (CO_2_) to the bulk material through interconnected porosity. Thus, sintering phenomena must be avoided as much as possible to maintain sufficiently high oxidation rates and thereby high CO yields.

**Figure 16:**
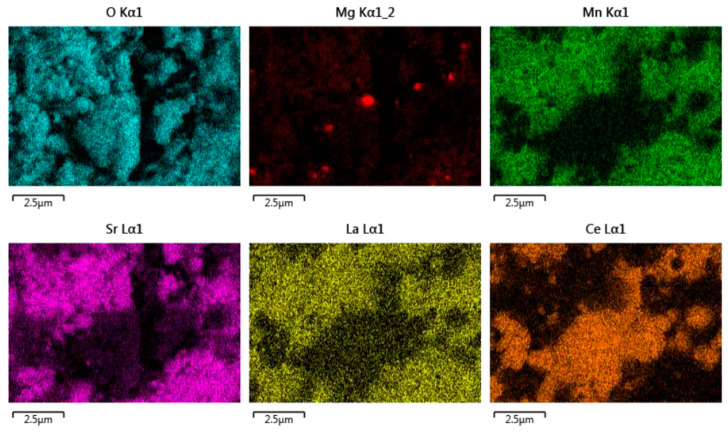
EDS analysis of CeO_2_/LSMMg (50/50 wt%) dual-phase foam after calcination at 1400 °C.

The EDS analysis (Figure 16) shows that the two distinct phases remain, but it also reveals that the magnesium has migrated from the perovskite phase to form segregated spots on the ceria grains.

**Figure 17:**
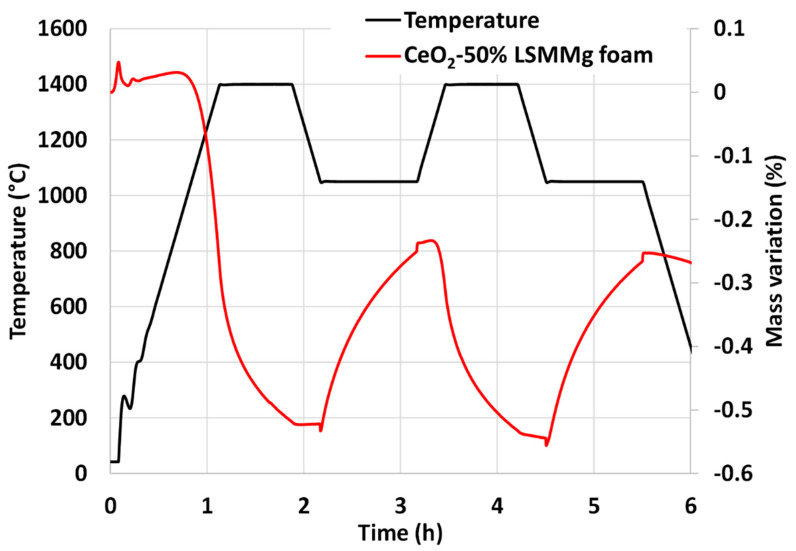
TGA of CeO_2_/LSMMg (50/50 wt%) dual-phase foam during two consecutive CO_2_-splitting cycles.

The CeO_2_/LSMMg foam was analyzed by TGA during two consecutive cycles (Figure 17). The releases of oxygen during the reduction steps are 173 µmol/g and 96 µmol/g and the CO production yields reach 176 µmol/g and 180 µmol/g. The production of CO with the foam is thus consistent with the production obtained with the mixture of powders without shaping under the same cycling conditions.

**Figure 18:**
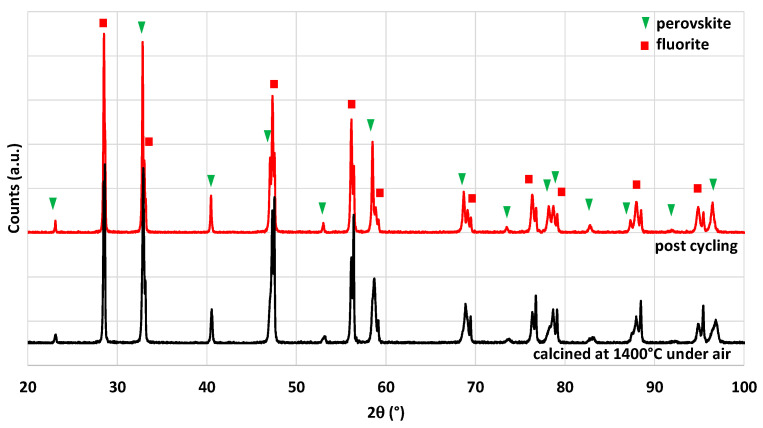
XRD patterns of CeO_2_/LSMMg (50/50 wt%) dual-phase foam after calcination at 1400 °C and after two consecutive thermochemical CO_2_-splitting cycles.

XRD patterns of the crushed foam calcined at 1400 °C and after two thermochemical cycles are compared in Figure 18. The two phases corresponding to CeO_2_ and LSMMg are observed without any by-product before and after the thermochemical cycles, which confirms the stability of the material and the absence of reaction between phases.

**Figure 19:**
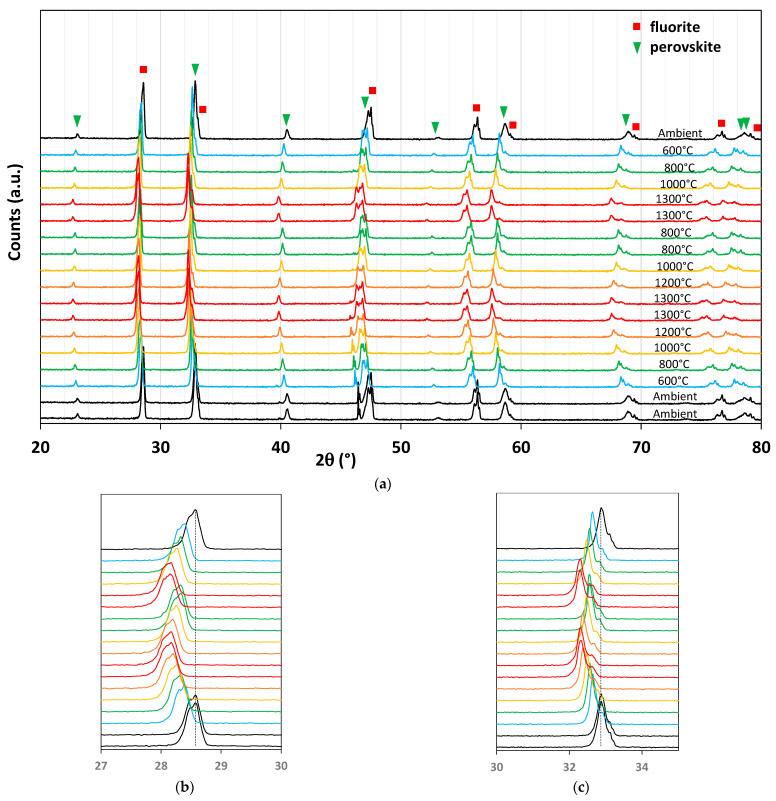
XRD analysis of CeO_2_/LSMMg (50/50 wt%) dual-phase foam during two consecutive CO_2_-splitting cycles (**a**), zoom on a ceria peak (**b**), and zoom on a perovskite peak (**c**).

In situ temperature-programed XRD analysis during two thermochemical cycles was then performed. Figure 19a shows the superimposition of the diffraction patterns of the ground foam for the different temperatures. From bottom to top, the material was heated from room temperature to 1300 °C under nitrogen, then the temperature decreased to 800 °C, at which CO_2_ was injected into the analysis chamber, followed by a similar second cycle. Diffraction peaks are shifted to low angles during reduction steps and to high angles during cooling and CO_2_ exposure. These peak shifts correspond to the reduction and oxidation of the two materials. The beneficial synergistic effect described in previous work on La_0.65_Sr_0.35_MnO_3_–CeO_2_ composite [43] was not observed in this study. The O_2_ release and CO production yields are lower than those of pure LSMMg, and the effect of the oxygen diffusion channel by ceria was not observed. Indeed, Bork et al. [43] reported a shift of the LSM XRD peak to a lower angle upon cooling after the reduction step (corresponding to LSM reduction with oxygen transfer inducing simultaneous re-oxidation of ceria). They concluded that the ceria only played the role of oxygen diffusion channel, but this was not the case in this study as both phases were redox-active (Figure 19b,c).

In summary, to conclude this section on the CeO_2_/LSMMg (50/50) composite material, no synergistic effect was observed and CO production was not enhanced compared to the pure LSMMg phase. However, fuel production performance was improved over pure ceria despite slower oxidation rates.

## 3. Materials and Methods

Several synthesis methods were employed to obtain the different oxides investigated in this study. Different methods were chosen mainly to compare the redox performance with the materials synthesized in other studies based on the same methods.

Coprecipitation of hydroxides has been used for the synthesis of nickel ferrite and Ce_0.9_Fe_0.1_O_2_ oxides. Nitrates precursors (Ce(NO_3_)_3_.6H_2_O, purity 99.5%, Ni(NO_3_)_2_.6H_2_O, purity 99.5%, and Fe(NO_3_)_3_.9H_2_O, purity ≥98% from Sigma-Aldrich) were weighted to adapt to the desired stoichiometric content and dissolved in distilled water by stirring. Polyethylene glycol (purity ≥99%) was added to the solution under stirring to improve the final powder morphology. Sodium hydroxide (NaOH) was then poured into the solution until the pH value reached 11. The metallic cations were hydrolyzed and formed a precipitate of metal hydroxides. The precipitate was filtered and washed several times until the pH of the rinsing solution decreased to 7. The precipitate was then dried in an oven at 80 °C overnight and calcined at 1000 °C for 1 h. The solid was ground in a mortar and calcined at 1400 °C (or 1300 °C for Ce_0.9_Fe_0.1_O_2_ to alleviate sintering) to form the final oxide.

The second synthesis method employed was the modified Pechini synthesis described elsewhere [48]. Briefly, metallic nitrate precursors were dissolved in distilled water by stirring for 10 min. The citric acid (purity ≥99.5%) was then added to the solution under stirring with a molar concentration twice the cations concentration (n_CA_= 2 × n_cations_). Ethylene glycol (purity ≥99%) was then poured into the solution with a molar concentration four times the cations concentration (n_EG_ = 4 × n_cations_). The solution was stirred and heated up to 90 °C until gelation (brownish color). The gel was then heated in a furnace at 250 °C for 2 h. The resulting powder was crushed in an agate mortar and calcined in air at 1000 °C for 1 h. To avoid any sintering effect during the thermal analysis, the oxides were then calcined and stabilized in air for 1 h at either 1300 °C or 1400 °C corresponding to the maximum temperature of the thermogravimetric analysis (TGA).

Another synthesis technique employed was the evaporation-to-dryness method used to synthesize Ce_0.2_Sr_1.8_MnO_4_ [37]. The metal nitrate precursors were dissolved in distilled water by stirring. The solution was then heated until complete evaporation of the water and the solid residue was first calcined at 1100 °C for 2 h, then at 1400 °C for 10 h.

The last method employed was a solid-state synthesis using a vibrating milling apparatus (mixer mill MM400 Retsch). To synthesize Ce_0.2_Sr_1.8_MnO_4_, the selected precursors were CeO_2_ commercial powder (99.9%, <5 µm, Sigma-Aldrich), MnO (≥99%) commercial powder, and SrCO_3_ (≥99.9%). The precursors were weighed and placed in the mixer mill with water (37% wt). The paste was milled for 30 min at a frequency of 30 Hz. The paste obtained was dried at 110 °C for 6 h and calcined at 1400 °C for 3 h.

The reticulated foams were obtained from the corresponding powders by the replication method already described elsewhere [49]. The oxide powders were ground together in the case of dual-phase materials in a mortar and used in the slurry formulation. The different oxides were weighed and poured into distilled water together with the porogen (carbon fibers Sigrafil^®^ M80), the dispersant (Dolapix^®^), and the binder (polyvinyl alcohol, PVA, Mowiol^®^ 4-88). The aqueous slurry was then used to coat/impregnate a sacrificial polyurethane (PU, Bulpren) foam (10 ppi, pore per inch) serving as a template. The PU foam was immersed in the slurry and dried overnight. The as-impregnated foam was then slowly heated up to 1000 °C (at 2.5 °C/min) to remove all the organic components from the material and annealed at 1400 °C for 3 h to achieve sintering which provides mechanical resistance to the ceramic foam.

The structure and microstructure of the prepared materials were fully characterized with the following series of techniques.

The crystalline structure was studied by X-ray diffraction (XRD) using a Panalytical X’PERT PRO diffractometer with the Cu Kα radiation (αCu = 0.15406 nm, angular range = 20–80°, 2θ, tube current 20 mA, potential 40 kV).

High-temperature X-ray diffractometer (HT XRD—Empyrean Panalytical with Anton-Paar HTK1 chamber) equipped with Cu Kα radiation was used for in situ investigation of phase reactivity/transitions during redox cycling (tube current 20 mA, potential 45 kV, angular range = 20–80°, 2θ, angle variation 0.02626°/s). The powder was heated up to 1300 or 1400 °C under N_2_ flow for the reduction step and cooled down to either 1000 °C or 800 °C for the oxidation step with CO_2_ injection.

The morphology of the materials was observed with a Field Emission Scanning Electron Microscope (FESEM–Hitachi S4800) used to examine the microstructure of foams.

A chemical elementary cartography was realized by EDS analysis (Energy Dispersive X-ray Spectroscopy) to estimate the chemical composition and to observe cations distribution (surface mapping) in the material. Analyses were performed using a Zeiss Sigma 300. The accelerating voltage used was 15 keV.

To complete the materials characterization, their thermochemical activity during two-step CO_2_-splitting cycles was investigated. Thermogravimetric analysis (TGA, SETARAM Setsys Evo 1750) was used to measure the mass variations (amount of oxygen exchanged) associated with the reduction and oxidation steps of thermochemical cycles. About 100 mg of material (exact mass determined with a high precision balance) was introduced in a platinum crucible hung to the microbalance with platinum suspensions and placed inside the furnace chamber. Residual air in the furnace chamber was eliminated by preliminary pumping to operate in an inert atmosphere during the reduction step with a low oxygen partial pressure (p_O2_ ~ 10^−5^ bar). The sample was then heated under a flow of argon (99.999% purity, <2 ppm of O_2_, 20 mL/min) with a heating rate of 20 °C/min up to the selected set-point temperature, and the variation in mass was recorded continuously. The successive steps of the cycles were carried out at different temperatures (typically 1400 °C or 1300 °C dwelled for 45 min during the reduction step and 1050 °C dwelled for 1 h during the CO_2_-splitting step), and CO_2_ (99.995% purity) was injected during the oxidation step (with a mole fraction of 50% CO_2_ in argon and with a CO_2_ flowrate of 10 mL/min). The same cycling conditions were applied for all materials for comparison purposes. The maximum cycle temperature was a good compromise because it was high enough to guarantee sufficient reduction yield while maintaining the thermal stability of the materials. Blank run correction (using an empty crucible) was performed to correct the small baseline mass drift during non-isothermal heating caused by thermal gas expansion and buoyancy effects on the crucible.

The mass variations associated with the temperature-programmed cycles correspond to the oxygen release during reduction (in the form of O_2_) and the oxygen uptake (in the form of O atoms) during CO_2_-splitting (equivalent to the CO produced), which allows calculating both the reduction yield and the CO production yield. Accordingly, the O_2_ and CO production yields (expressed in mol/g_oxide_) are calculated as follows:

(3) $$n_{O_{2}}={\Delta m}_{\mathrm{red}}/\left(M_{O_{2}}\cdot m_{\mathrm{oxide}}\right)$$

(4) $$n_{\mathrm{CO}}={\Delta m}_{\mathrm{ox}}/\left(M_{O}\cdot m_{\mathrm{oxide}}\right)$$

where Δm is the sample mass variation measured during the reduction or the oxidation step (g), M is the molecular weight of O_2_ (32 g/mol) or O (16 g/mol), and m_oxide_ is the sample mass loaded in the crucible (g).

## 4. Conclusions

This study investigated different classes of redox-active compounds based on spinel ferrite, fluorite, and perovskite oxide structures for the generation of synthetic solar fuels via two-step thermochemical cycles. The process ultimately aims to use concentrated solar energy as a high-temperature process heating source for thermochemical reactions. First, state-of-the-art redox materials consisting of Ni-ferrite and ceria were shaped as reticulated foams to compare their thermochemical performance during cycling with a reduction step temperature of 1400 °C and a re-oxidation step under CO_2_ (50% mole fraction) at 1050 °C. As expected, the NiFe_2_O_4_ foam produced more CO than its ceria counterpart, at the expense of slower kinetics during the oxidation step. Other single-phase oxides were then considered (including Ce_0.9_Fe_0.1_O_2_, Ca_0.5_Ce_0.5_MnO_3_, Ce_0.2_Sr_1.8_MnO_4_, and Sm_0.6_Ca_0.4_Mn_0.8_Al_0.2_O_3_) as they were previously identified in separate studies as highly reactive materials for CO_2_ splitting, with reported performance significantly superior to that of the benchmark ceria or lanthanum manganite perovskites. Nevertheless, these results were not confirmed in this work as the materials showed limited fuel production and lower overall redox performance in comparison with La_0.5_Sr_0.5_Mn_0.9_Mg_0.1_O_3_, due to sintering effects or kinetic limitations during oxidation which were never highlighted before.

In order to improve the performance of materials, dual-phase composites were finally considered, in particular ceria/ferrite and ceria/perovskite composites. The addition of NiFe_2_O_4_ to ceria did not prove to be beneficial in improving redox performance. Among the perovskites considered (La_0.5_Sr_0.5_Mn_0.9_Mg_0.1_O_3_, Ca_0.5_Sr_0.5_MnO_3_, Y_0.5_Sr_0.5_MnO_3_), it was shown that the addition of 50 wt% of La_0.5_Sr_0.5_Mn_0.9_Mg_0.1_O_3_ improved fuel yield at the expense of slower oxidation kinetics compared to pure ceria. Interestingly, the same composite formulation shaped as a reticulated foam with dual-scale porosity maintained its fuel production capacity (~180 µmol/g) without altering the reaction kinetics. However, no synergistic effect between the two phases was demonstrated.

The consideration of composite materials remains a promising option to improve the oxygen-exchange capacity of redox materials for the production of renewable fuels. When mixing redox-active phases, synergistic effects resulting from bulk-to-surface oxygen transfer in the composite materials are expected, by which the redox properties could be modified and ultimately the fuel productivity could be improved. The expected beneficial effects of dual-phase composites therefore deserve to be thoroughly assessed and further demonstrated.

## Figures and Tables

**Table 1:** Summary of O_2_ release and CO production yields, along with CO peak production rates during two consecutive thermochemical cycles with the considered series of perovskite powders (note that the reduction step for Ca_0.5_Ce_0.5_MnO_3_ was carried out at 1300 °C).

Perovskite Composition	Synthesis Method	1st Cycle			2nd Cycle		
		O_2_ Yield	CO Yield	CO Peak Production Rate	O_2_ Yield	CO Yield	CO Peak Production Rate
		(µmol/g)	(µmol/g)	(µmol/min.g)	(µmol/g)	(µmol/g)	(µmol/min.g)
La_0.5_Sr_0.5_Mn_0.9_Mg_0.1_O_3_	Pechini	254	246	15.3	203	249	12.7
Ce_0.2_Sr_1.8_MnO_4_	Evaporation-to-dryness	201	219	-	114	224	-
Ce_0.2_Sr_1.8_MnO_4_	Mecano-synthesis	197	184	15.7	121	199	6.9
Ca_0.5_Ce_0.5_MnO_3_	Pechini	260 (1300 °C)	53	1.6	48 (1300 °C)	76	2.2
Sm_0.6_Ca_0.4_Mn_0.8_Al_0.2_O_3_	Pechini	206	213	25.4	180	212	6.4

**Table 2:** O_2_ and CO production yields, along with CO peak production rates during two-consecutive thermochemical cycles with the dual-phase material CeO_2_/NiFe_2_O_4_ (50/50 wt%) compared to pristine ceria and Ni-ferrite.

Powder Composition	1st Cycle			2nd Cycle		
	O_2_ Yield	CO Yield	CO Peak Production Rate	O_2_ Yield	CO Yield	CO Peak Production Rate
	(µmol/g)	(µmol/g)	(µmol/min.g)	(µmol/g)	(µmol/g)	(µmol/min.g)
CeO_2_/NiFe_2_O_4_ (50/50 wt%)	226	85	7.5	76	104	9.1
CeO_2_	55	95	39.5	54	98	43.7
NiFe_2_O_4_	201	172	6.2	106	158	6.2

**Table 3:** O_2_ and CO production yields, along with CO peak production rates during two thermochemical cycles in TGA for a series of dual-phase powder compositions, in comparison with single CeO_2_ and LSMMg powders.

Powder Composition	1st Cycle			2nd Cycle		
	O_2_ Yield	CO Yield	CO Peak Production Rate	O_2_ Yield	CO Yield	CO Peak Production Rate
	(µmol/g)	(µmol/g)	(µmol/min.g)	(µmol/g)	(µmol/g)	(µmol/min.g)
CeO_2_	55	95	39.5	54	98	43.7
LSMMg	254	246	15.3	203	249	12.7
CeO_2_-10% YSM	117	123	15.0	71	129	16.7
CeO_2_-10% LSMMg	45	83	14.1	57	98	12.3
CeO_2_-50% LSMMg	120	178	25.7	124	163	6.6
CeO_2_-10% CSM	172	129	13.1	74	137	15.5
CeO_2_-50% CSM	652	151	5.1	93	168	5.8

## Data Availability

The data presented in this study are available on request from the corresponding author.
